# A structured, behavioural science approach to the preparation of antimicrobial stewardship interventions for companion animal veterinarians

**DOI:** 10.1002/vetr.6016

**Published:** 2025-12-04

**Authors:** Ben Walker, Dan O'Neill, Huw Stacey, Dave Brodbelt

**Affiliations:** ^1^ Veterinary Epidemiology, Economics and Public Health Group, Department of Pathobiology and Population Sciences The Royal Veterinary College Hatfield UK; ^2^ Tamar Vets Bude UK

**Keywords:** antimicrobial prescription, antimicrobial stewardship, behavioural science, companion animals

## Abstract

**Background:**

A structured, behavioural science approach was adopted to develop antimicrobial stewardship interventions in UK first‐opinion companion animal veterinary practice. The objectives were to identify behavioural targets for intervention and to understand how practice contexts influence these behaviours.

**Methods:**

Behavioural targets were selected by expert collaborators from a longlist prepared by the lead author. Five veterinary practices were recruited through opportunistic sampling. The lead author shadowed 17 consulting veterinarians, recording observations and informal interviews. Common themes were identified through a combined deductive and inductive approach to describe barriers to and enablers of the behavioural targets.

**Results:**

The behavioural targets were that veterinarians should: (1) document why they prescribed the highest‐priority antimicrobials, (2) perform cytology, (3) prescribe alternative pharmaceutical interventions, and (4) provide advice and reassurance. Important barriers were that veterinarians were unfamiliar with less critically important antimicrobials for cats and non‐prescription topical chlorhexidine products.

**Limitations:**

A single researcher was responsible for constructing the longlist of behavioural targets, visiting practices, collecting data, and coding observations and quotations. The behavioural targets were complex and data saturation was unlikely to have been achieved.

**Conclusions:**

Choosing and exploring behavioural targets in a structured manner identified clear and specific targets for intervention.

## INTRODUCTION

Promoting antimicrobial stewardship in both the human and veterinary spheres is considered a critical step to limit the threat of antimicrobial resistance. Antimicrobial stewardship describes prescribers using antimicrobials responsibly and involves promoting actions that balance both the animal's need for appropriate treatment and the longer‐term societal need for sustained access to effective therapies.^1^

In the UK companion animal veterinary sector, key stakeholders, including the UK government,^2^ Royal College of Veterinary Surgeons (RCVS),^3^ British Veterinary Association^4^ and British Small Animal Veterinary Association,^5^ have all recognised the importance of antimicrobial stewardship in veterinary practice. One aspect of antimicrobial stewardship commonly promoted, as part of the broader goal of improved appropriateness of antimicrobial usage, is the aim of optimising both overall systemic antimicrobial prescribing and that of the antimicrobials most important to human medicine (Table 1): the World Health Organization classification highest priority critically important antimicrobials (HPCIAs),^6^ and the broadly equivalent European Medicines Agency Category B ‘Restrict’ and the WOAH category, veterinary critically important antimicrobial agents.^7^, ^8^

**TABLE 1:** Common companion animal veterinary antimicrobials and their World Health Organization categories^6^

Category	Class	Examples
Highest priority critically important antimicrobials	Third and fourth‐generation cephalosporins	Cefovecin
	Macrolides	Erythromycin Tylosin
	Quinolones	Enrofloxacin Marbofloxacin Pradofloxacin
Critically important antimicrobials	Aminoglycosides	Gentamycin Streptomycin
	Aminopenicillins	Amoxicillin
	Aminopenicillins with beta‐lactamase inhibitors	Amoxicillin‒clavulanate
Highly important antimicrobials	Amphenicols	Chloramphenicol
	First and second‐generation cephalosporins	Cefalexin Cefuroxime
	Lincosamides	Clindamycin
	Narrow‐spectrum penicillin	Benzylpenicillin (Penicillin G) Phenoxymethylpenicillin (Penicillin V)
	Sulfonamides	Trimethoprim sulfadiazine
	Tetracyclines	Oxytetracycline Doxycycline
Important antimicrobials	Nitrofurans	Nitrofurantoin
	Nitroimidazoles	Metronidazole

Several studies over recent years have assessed the impact on antimicrobial usage of a range of interventions, such as providing training to veterinarians on prescribing guidelines;^9^ providing audit and feedback alongside training;^9^, ^10^ or more complex packages of interventions.^11^, ^12^ However, their outcomes have been inconsistent. Most studies showed reductions in overall systemic antimicrobial use,^9^, ^11^, ^12^, ^13^ although one found an increase.^10^ Some showed reductions in the absolute or relative prescribing of HPCIAs^9^, ^10^, ^12^ while others found minimal change^11^ or even increases.^14^ Where absolute or relative HPCIA prescription dropped, there was usually a corresponding increase in prescription of amoxicillin‒clavulanate,^10^, ^12^, ^13^ which is itself classified as a critically important antimicrobial (CIA).

One possible explanation for these inconsistencies is the design of the interventions. None of these earlier studies applied a structured approach to intervention design with clearly specified behavioural targets.

In response, the current study aimed to adopt a structured, behavioural science approach^15^ to identify behaviours that could be targets for interventions. We hoped to identify interventions more likely to be taken up by both practices and veterinarians and to limit unintended consequences by proposing interventions based on evidence from stakeholders within practice.^16^ Our objectives were to answer two questions. First, which veterinary behaviours are amenable to antimicrobial stewardship interventions? Second, how do current veterinary practice contexts influence these behaviours?

## MATERIALS AND METHODS

### Choosing behavioural targets

We used existing antimicrobial usage guidelines^4^, ^5^, ^17^, ^18^ and resources developed by a collaborating veterinary practice group to produce a summary of various recommendations for antimicrobial usage (Tables S1–S3).

In the UK, when selecting which antimicrobial to prescribe, veterinarians are not required to follow a specific set of antimicrobial prescribing rules, but are expected to ensure that ‘when using antimicrobials they do so responsibly’.^3^ However, there is no single agreed‐upon rule that defines responsible antimicrobial use. We therefore took guideline recommendations where consensus was clear across the various guidelines, alongside conclusions from previous qualitative literature^19^, ^20^ and our clinical experience to develop conceptual process maps showing how we believe antimicrobials are prescribed or not prescribed in the typical UK companion animal veterinary clinical environment.

**FIGURE 1:**
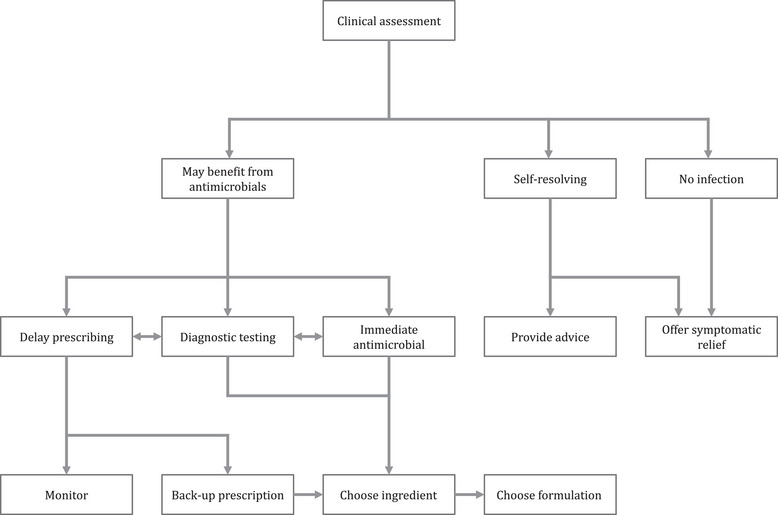
Conceptual map of how antimicrobials are prescribed in veterinary clinical practice

The maps focused on key actions and decisions that veterinarians make following a clinical assessment, from their diagnosis, to deciding a management strategy, to implementing a specific therapeutic plan. Using these maps and building on the action, actor, context, target and time framework,^21^ we proposed a longlist series of specific behaviours that offered potential intervention targets (Figures S1–S3). We chose to limit our longlist to behaviours directly influencing veterinarians’ decisions to prescribe systemic antimicrobials at all. Our final process map (Figure 1) gave us a longlist of 13 behavioural targets (Table 2).

**TABLE 2:** Candidate behavioural targets assessed for suitability for antimicrobial stewardship intervention by study authors and collaborators

			Behavioural target	Author and collaborator comments		
				Acceptability	Effectiveness	Risk of adverse events
Longlist	Shortlist	Final selection	Veterinarians should document the reason for prescribing an HPCIA instead of a lower priority antimicrobial.	High	Moderate. Likely to see a move to other systemic antimicrobials instead	Low
			Veterinarians should perform cytology for cases presenting with pyoderma and otitis externa prior to starting and ending treatment.	High	High	Low
			Veterinarians should offer alternative pharmaceutical treatments such as topical treatments for pyoderma and otitis externa, and non‐steroidal anti‐inflammatory drugs for pyrexia, feline lower urinary tract disease and upper respiratory tract disease.	High	High	Low
			Veterinarians should provide advice and reassurance for self‐limiting conditions such as acute diarrhoea, vomiting or acute upper respiratory disease.	Moderately high	High	Low
			Veterinarians should adopt a ‘wait and see’ approach to stable patients with non‐specific illness.	Moderately high	Moderate	Moderate, if delaying treatment in sick patients
			Veterinarians should prescribe perioperative antimicrobials where implants are used, the surgery is over 90 minutes duration, for dirty or contaminated procedures, or for sick patients.	High	Low, as practices are largely doing this already	Low
			Veterinarians should admit pets with severe vomiting, haemorrhagic diarrhoea and dental abscesses without prescribing systemic antimicrobials.	High	Low, as there are few cases to whom this applied	Low
			Veterinarians should prescribe HPCIAs only where culture and sensitivity indicate they are required	Low	High	Moderate, main risk is withholding critical drugs where culture and sensitivity are not possible
			Veterinarians should perform culture and sensitivity in cases where: topical treatment has failed, a course duration of longer than 1 week is needed, there is recurring infection, rods are visualised on cytology, HPCIA are prescribed, the patient is systemically ill, or the diagnosis is uncertain.	Low, as culture and sensitivity are rare	Low, there are few cases to which this applies	Low
			Veterinarians should follow their local policy when one is present.	Moderate	Low, as few practices have local prescribing policies	Low, but would depend on the policies
			Veterinarians should document the reason for prescription for all systemic antimicrobial prescriptions.	Low, as it is a big demand	Uncertain	Low
			Veterinarians should perform haematology in all unstable or systemically unwell patients and all immunocompromised patients prior to antimicrobial prescription.	Low, due to cost of the procedure	Low, as it applies to few cases	Low
			Veterinarians should perform diagnostic imaging in cases presenting with lower respiratory disease prior to antimicrobial prescription.	Low, due to cost of the procedure	Low, as it applies to few cases	Low, but exposes animals to anaesthetic

Abbreviation: HPCIA, highest priority critically important antimicrobial.

Two rounds of discussion between the authors and their collaborators were undertaken to first select a shortlist of 7–10 behaviours from the original longlist and finally to select 3–5 behavioural targets. These discussions considered the likelihood that interventions targeting the behaviour would be accepted by veterinarians, and how large an impact veterinarians adopting the behaviours were likely to have on the overall reduction in unnecessary antimicrobial usage and that of HPCIAs. Finally, we explored the nature and probability of unintended consequences to veterinarians, their patients, and their clients from targeting these behaviours.

For the initial shortlisting of behavioural targets (round one), alongside the study authors, we invited a non‐clinical post‐doctoral researcher (A.T.) who had previously researched opportunities and challenges around antimicrobial stewardship initiatives.^19^ For the final selection of our behaviour targets (round two), we invited our other four expert collaborators to participate alongside the participants from the first group. Two of these four collaborators (D.S. and D.L.) with relevant backgrounds in antimicrobial stewardship, epidemiological research and veterinary dermatology were able to attend.

### Understanding practice contexts

We recruited practices from one practice group using convenience sampling based on their proximity to the lead author (B.W., a veterinary epidemiologist who previously worked in first‐opinion companion animal practice for 8 years). Although the practice group provides some resources and information to practices, individual practices are run as clinically independent entities. We aimed to recruit 3–5 practices and for the lead author to spend 2 days in each practice. We emailed practice owners sequentially asking them to participate based on their practice's location and convenience to the lead researcher until we had successfully recruited a maximum of five practices, as 10 days was the maximum amount of time available to the researcher. We aimed to observe every veterinarian who performed consultations during the 2 days spent in each practice.

The lead author shadowed consulting veterinarians, observing their environments, their behaviours, and their interactions with other staff and clients. He asked questions and conducted informal interviews when the opportunities arose to better understand their behaviours and decision making. Field notes were recorded by hand while in the practices and later transcribed into NVivo^22^ for coding. Our consent forms had a pre‐entered, unique, random, five‐digit token that we used as an anonymous identifier for participating veterinarians.

We adopted a deductive approach to categorise observations and quotations into theoretical domains,^23^ grouped by behavioural targets. Next, we used an inductive approach to produce a narrative summary showing common themes.

### Behavioural diagnoses

Finally, for each of our behavioural targets, we extracted the relevant insights from our inductive approach to summarise the barriers to and enablers of our behavioural targets as behavioural diagnoses.

## RESULTS

### Choosing behavioural targets

The longlist of behavioural targets is reported in Table 2, which also reports which behavioural targets were eliminated during the two rounds of discussion. The final selection was based on those targets the authors and collaborators judged overall to have the greatest likely acceptability and effectiveness with the lowest risk of unintended consequences. The five selected behavioural targets were as follows:

1. Veterinarians should document their reason for prescribing the highest‐priority, critically important antimicrobials.
2. Veterinarians should perform cytology in veterinary patients presenting with pyoderma and otitis externa to determine the need for systemic antimicrobial therapy and the choice of antimicrobial.
3. Veterinarians should prescribe alternative treatments, including topical treatment for superficial pyoderma, and NSAIDs for pyrexia, feline lower urinary tract disease and acute upper respiratory disease.
4. Veterinarians should provide advice and reassurance but without prescribing a systemic antimicrobial for the generally self‐limiting conditions of acute diarrhoea, vomiting and acute upper respiratory disease.
5. Veterinarians should adopt a ‘wait‐and‐see’ or a delayed prescribing approach to antimicrobial prescribing in cases of non‐specific illness with an uncertain diagnosis, where the patient is stable.

The round two panel members considered that behaviours 4 and 5 would have considerable overlap in their theoretical domains, so we considered them together as a single behavioural target of providing advice and reassurance.

### Understanding practice contexts

We recruited five practices from seven initially contacted, all in the south of England. The two practices that declined to participate reported staffing problems as their reason for declining. Of the five practices that were visited, one was staffed by one veterinarian, one by two veterinarians, and the other three had three or more veterinarians. All five practices were also experiencing staffing limitations and had stopped offering Sunday appointments as a result. In conjunction with being part of a practice group, one practice was owned by a single veterinarian, two practices by two veterinarians, one practice by two veterinarians and a veterinary nurse, and one practice by three veterinarians.

We recruited every veterinarian who performed consultations during the 2 days of observation at each practice visited, providing observations and quotations from 17 veterinarians in total (nine males and eight females).

The main observations grouped by theoretical domain and behaviour target are shown in Table 3. We identified four broad themes that we can summarise as follows: attitudes towards antimicrobials, alternatives to systemic antimicrobials, client expectations and confidence with cytology.

**TABLE 3:** Observations of behavioural targets for antimicrobial interventions by theoretical domain^23^

Theoretical domain	Documenting HPCIA use	Alternative therapeutics	Cytology	Advise and reassure
Knowledge	Veterinarians knew that HPCIAs were ‘high tier’ antimicrobials but did not consistently know why. Most veterinarians were unable to correctly identify narrow‐spectrum antimicrobials. Veterinarians had limited knowledge of cat‐friendly treatment options (no‐rinse topical chlorhexidine, antimicrobial liquids and pastes), so cefovecin was often seen as inevitable if tablets were declined.	Veterinarians were aware of guidelines and the need to use alternatives. There was a good knowledge of alternative therapeutics for many common illnesses. However, they were unaware of many topical chlorhexidine products for superficial pyoderma.	Veterinarians knew that cytology was recommended for pyoderma and otitis but did not know how the findings might guide treatment decisions.	Veterinarians knew that antimicrobials were not indicated in acute diarrhoea but generally underestimated the typical expected duration of signs, meaning antimicrobials were still given. Knowledge around feline lower urinary tract disease and rhinitis was more variable with some veterinarians reporting cefovecin use for cystitis.
Skills	No themes emerged in this domain	No themes emerged in this domain	A small number of veterinarians were confident with cytology and performed it routinely as part of their consultations. Some claimed confidence but did not perform it, suggesting they struggled to perform it efficiently. Most were not confident performing cytology and did not perform it routinely.	Veterinarians were not confident providing advice as the sole outcome of a consultation.
Social/professional role and identity	Most veterinarians felt that their use of cefovecin would be frowned upon. Veterinarians in low‐prescribing practices were proud of the fact they prescribed less than others in the group. One veterinarian voiced concerns that physicians and large animal veterinarians were not focussing on the problem and their efforts may be a waste of time.	No themes emerged in this domain	Many veterinarians felt that performing cytology was better suited to the veterinary nurse role.	Veterinarians were frequently worried about complaints or a loss of confidence from clients if they misjudged when to reassure them. Veterinarians worried that they were not as well respected as they used to be.
Beliefs about capabilities	No themes emerged in this domain	No themes emerged in this domain	Most veterinarians did not believe they could efficiently perform cytology as part of their standard consultations.	No themes emerged in this domain
Optimism	Many veterinarians voiced concern that the animal would not get better without providing an antimicrobial even when they were aware of evidence	that it was unnecessary.	Some veterinarians were concerned that if they offered cytology it would be declined anyway due to its cost.	No themes emerged in this domain
Beliefs about consequences	Many veterinarians believed that their clients would be unable to adequately treat their cats if they prescribed tablets. This was often an implicit (undocumented) reason for prescribing cefovecin.	Veterinarians often accepted the alternative therapeutic recommendations but frequently prescribed them alongside a systemic antimicrobial. They were concerned that the animal would have a worse recovery without it.	Many veterinarians thought that performing cytology would not change their treatment plan.	Veterinarians often believed that clients expected a prescription and would complain if one was not provided. Some veterinarians were confident that animals would recover well without prescriptions, although some believed they would be risking a longer or poorer recovery. One veterinarian was worried that clients might not monitor their pet's illness as seriously if no antimicrobial had been prescribed.
Reinforcement	No themes emerged in this domain	Veterinarians reported that having seen successful outcomes after using antimicrobials they were reluctant to change their prescribing behaviours.	No themes emerged in this domain	Veterinarians reported that having seen successful outcomes after using antimicrobials they were reluctant to change their prescribing behaviours.
Intentions	One practice owner reported having policies around course lengths, but not around therapeutic selection.	No themes emerged in this domain	No themes emerged in this domain	No themes emerged in this domain
Goals	Most practice owners expected their cefovecin use to decrease over time. The only owner who measured their use aimed to be anywhere below the group average.	No themes emerged in this domain	No themes emerged in this domain	No themes emerged in this domain.
Memory, attention and decision processes	No themes emerged in this domain	No themes emerged in this domain	No themes emerged in this domain	No themes emerged in this domain
Environmental context and resources	Most practices did not have many alternatives to cefovecin in their dispensaries. Amoxicillin suspension for injection was only available in one practice, clindamycin liquid and doxycycline pastes were rarely present, and there were few commercial chlorhexidine preparations. One practice had no dental radiography. The veterinarian atomised roots that fractured during extractions and prescribed cefovecin as a precaution against post‐operative root abscesses.	Many practices kept a limited selection of topical skin products on the shelves for use in pyoderma. A prescription shampoo and some creams for focal pyoderma were generally available. Most practices kept a 2%–4% surgical chlorhexidine scrub on the shelves that was prescribed for pyoderma at 1:5–1:10 dilutions.	Every practice had a microscope and stains available. These were generally well‐maintained, although one had oil on the 40× objective lens. Some practices did not have a clear fee for cytology. The fee varied from £20 to £50. Most veterinarians reported having inadequate time to perform cytology. This was true for veterinarians allocated 10, 15 and 20 minutes. Veterinarians who were confident performing cytology did not voice this concern.	No practice provided a local prescribing policy or any informational materials to assist or support their veterinarians in not prescribing an antimicrobial.
Social influences	One practice reported switching to amoxicillin long‐acting injections instead of cefovecin following a group audit that revealed high use. Veterinarians reported not documenting the reason for cefovecin use as they did not feel their usage was ‘off‐label’.	Veterinarians reported changing their behaviours due to influences from senior colleagues. In some practices that meant prescribing antimicrobials more, in others less.	No practice explicitly expected their veterinarians to perform cytology. One practice owner performed it confidently and routinely but seemed unaware that the rest of the team struggled.	Veterinarians were worried about giving advice that contradicted what the client had previously experienced.
Emotion	No themes emerged in this domain	No themes emerged in this domain	No themes emerged in this domain	Many veterinarians were worried they might misjudge whether an animal required antimicrobials.
Behavioural regulation	In general, veterinarians were keen to improve their clinical practice and had clinical governance systems to measure and change their behaviours. Most veterinarians had a clear understanding of their practice context and could explain and understand why their prescribing behaviours changed. One veterinarian was unable to reflect on why their cefovecin use had consistently increased over the preceding 8 months and was highly resistant to change. This was likely due to staffing problems at the clinic leading to stress and overwork.			

Abbreviation: HPCIA, highest priority critically important antimicrobial.

#### Attitudes towards antimicrobials

Veterinarians all knew of at least one antimicrobial stewardship campaign and were aware that antimicrobial usage guidelines existed. No veterinarian indicated that they used systemic antimicrobials for routine neutering or in the first few days of canine acute diarrhoea. Clinical audits^24^ were uncommonly carried out but the practice group provided the practices with summary reports of antimicrobial usage for the practice and the group as a whole. Some practices used these reports to inform change, but others used them to justify not prioritising reductions in unnecessary systemic antimicrobial usage.

I know our numbers are going down over time and we prescribe less than others in the group.

Veterinarians tended to categorise systemic antimicrobials as ‘low tier’ (or ‘first‐line’) and ‘high tier’. Both amoxicillin‒clavulanate (categorised as a critically important antimicrobial) and cefalexin (categorised as a highly important antimicrobial) were widely considered to be ‘low tier’ and they were sometimes prescribed in place of alternative non‐antimicrobial treatments.

I feel [cefalexin] works well for skin disease and it's a first‐line antibiotic. (Participant explaining why they didn't use chlorhexidine antiseptics)

Veterinarians knew that cefovecin and fluoroquinolones (both categorised as HPCIAs) were considered ‘high tier’ antimicrobials, although most could not explain what those statuses meant. No veterinarian was aware of the division between HPCIAs, critically important antimicrobials, highly important antimicrobials and important antimicrobials.

I know it's to do with resistance, but no more than that. If they tell me it's a worry then I'll believe them.

Veterinarians showed a desire to help animals and a concern that their veterinary patients may be harmed by withholding antimicrobial treatment. Veterinarians worried about misjudging the situation.

If I give [meloxicam] and [amoxicillin‒clavulanate] then I know tomorrow I will see him with a more normal temperature. If I just give [meloxicam] then maybe not. (Participant discussing a pyrexic, young adult cat)

Although none of the five practices had a formally documented local prescribing policy for antimicrobials, veterinarians reported local norms and prescribing habits from senior colleagues as influencing their own prescribing.

I trust [a senior veterinarian]. I'm not sure it's infected, but he says start on antibiotics so I think that should help.

#### Alternatives to systemic antimicrobials

Veterinarians had limited knowledge of ingredients and formulations for products that their practice did not stock. Cats tended to be given injectable cefovecin from a sense that it was the only practical option for superficial pyoderma because the practices stocked a limited range of systemic lower category antimicrobials, which did not include liquid formulations of clindamycin or cefalexin (both categorised as highly important antimicrobials), and topical chlorhexidine products.

I know [cefovecin] isn't ideal, but the owner can't manage tablets, so preds or other antibiotics aren't an option. (Participant discussing a cat with pyoderma)

In one practice, the team resolved this issue of reliance on cefovecin by stocking a long‐acting amoxicillin suspension for injection (categorised as a critically important antimicrobial) and veterinarians had switched to using that in many cases where they previously used cefovecin.

We used to use [cefovecin] far too much. Even cats that could have had tablets got it. Now we use [amoxicillin] LA and see them back or put them on oral courses.

For superficial pyoderma, veterinarians tended to use one of two topical antiseptics. Either a prescription, chlorhexidine/miconazole shampoo, or a chlorhexidine surgical scrub prescribed for use at below the recommended 2%–4% concentration. Few veterinarians reported knowing about commercially available foams, sprays, wipes and non‐prescription shampoos that clients could access. This meant that many veterinarians perceived few available options other than systemic antimicrobials.

#### Client expectations

Veterinarians worried their clients would become frustrated or angry if they did not resolve the problem at the first appointment.

People don't respect us as much as they used to … If I don't fix it immediately, they complain or won't see me again.

Veterinarians often believed that clients expected a therapeutic to be administered or dispensed and would be dissatisfied if the consultation was not coupled with medication.

Owners expect to leave with something. If they don't have a product, they'll wonder what they are paying the consult fee for.

#### Confidence with cytology

The management of skin disease was relevant to all four behavioural targets. However, using cytology to guide treatment decisions in cases of pyoderma and otitis externa had the widest range of factors influencing the four target behaviours.

Time limitations were commonly stated as a reason for not performing cytology.

I'm confident doing it and it is cheap for us to do and would bring in more money. I just don't have the time.

However, regardless of whether they had 10‐, 15‐ or 20‐minute consultations, veterinarians still reported time constraints as limiting their application of cytology in dermatology cases. Conversely, other veterinarians in these same practices who routinely performed cytology did not voice this time‐constraint complaint.

We have the longer consults so we can use the time to do this. We can do cytology in a consult.

Some veterinarians acknowledged their limitations and showed that they reflected on their expertise.

I'm not confident with skin cytology. Ears and lumps are ok, but I struggle to get good skin results.

Some veterinarians were concerned about the financial cost of the cytology procedure. The cost to the client of in‐house cytology varied across practices from £22 to £50. For otitis externa in particular, many veterinarians justified not using cytology with the belief they would prescribe the same topical medications regardless of cytological findings.

It costs and most people, they're paying for a consultation, and they are going to need some drops for the ears. They don't want to pay more when it won't change what we do.

### Behavioural diagnoses

Within the four behavioural targets, key observations from the practice interviews were identified and modifiable barriers and enablers are summarised as behavioural diagnoses.

#### Documenting reasons for prescribing HPCIAs

Veterinarians understood that HPCIA antimicrobials (e.g., cefovecin and fluoroquinolones) are ‘high tier’ and felt social pressure from veterinary professional bodies to use them less. However, they did not document their decision making for using an HPCIA instead of a lower tier antimicrobial because they often believed use of HPCIAs was the only option available. To see change here, veterinarians need to have, and be aware of, lower category or non‐antimicrobial alternatives, and they would need to believe that these alternatives will work.

#### Using cytology to guide pyoderma and otitis externa management

All the practices had the equipment needed to perform cytology. As a charged‐for service, practices can profit financially from promoting its use. To see change, veterinarians need to be confident in performing cytology, have sufficiently long consultations or other time periods to undertake cytology, believe cytology provides value for clients, and have clearly differentiated treatment pathways based on cytology results.

#### Prescribing alternative treatments

Veterinarians knew of many alternatives to systemic antimicrobial therapy for a variety of clinical presentations, for example, topical treatment for otitis externa and for uncomplicated cat bite abscesses, and believed that it was important to reduce unnecessary antimicrobial use. Superficial pyoderma was the exception where there was often limited knowledge of topical treatment options. Veterinarians were often not confident using alternative approaches without also providing an antimicrobial. To see change, veterinarians need greater understanding of what products are available, especially for topical management of superficial pyoderma. They need to believe that alternative therapeutics to antimicrobials can work well and be confident communicating that to their clients.

#### Providing advice and reassurance

Veterinarians recognised situations when watchful waiting was a clinically acceptable strategy. However, they were often worried about misjudging the situation and thereby harming their veterinary patients. They also worried about client expectations and were not confident communicating a watchful waiting strategy. To see change, veterinarians need to believe that watchful waiting can work well and be confident communicating that to their clients.

## DISCUSSION

The process of choosing and exploring behavioural targets in a structured manner in the current study helped to formulate a set of clear and specific targets for intervention.

Recent work in behavioural science has focused on the application of a structured approach to the design of interventions in health care with the intention of optimising the ability of these interventions to effect change.^15^, ^21^ In general, this structured approach advocates an intervention design that identifies the target behaviours based on evidence of practice gaps, uses a theoretical framework to establish the key barriers and enablers to be addressed and refines the main interventional components to reduce these barriers and encourage identified enablers.^15^, ^21^, ^23^ Such an approach was applied by French et al.^15^ to identify optimal management of human patients with acute lower back pain. An important element of this approach is the tailoring of interventions informed by qualitative research.^16^ In a European study of web‐based interventions to reduce inappropriate antimicrobial prescribing in human medicine, qualitative interviews of stakeholders were applied to facilitate successful uptake of the interventions across different healthcare systems and cultures.^25^ In the current study, individual veterinarian interviews and observations were invaluable in identifying how the proposed interventions might work and what were potential barriers and enablers.

It is reassuring that our behavioural findings are consistent with previous, more general, qualitative research into antimicrobial prescribing among companion animal veterinarians. Defensive prescribing,^20^, ^26^ beliefs about client expectations for antimicrobial treatment,^20^, ^26^, ^27^, ^28^, ^29^ and a deficiency of local prescribing policies^19^, ^26^, ^27^, ^30^ have been previously reported as key drivers to antimicrobial use and choice. So too have the understanding that cefovecin is a ‘high tier’ antimicrobial,^20^ the desire to slow antimicrobial resistance,^20^, ^26^, ^27^, ^28^ and veterinarians’ care for their patients.^20^ A recent Australian study has also highlighted the importance of developing strong communication skills to enable veterinarians to both withhold antimicrobials and maintain a positive relationship with their clients.^26^

It was interesting to observe that veterinarians who did not undertake cytology, frequently referred to a lack of time as a reason for not performing it. This was the case whether they had 10‐, 15‐ or 20‐minute consultations. In contrast, veterinarians who did use cytology did not consider time a major barrier to its use. Perceived challenges of time reported by some respondents may reflect individual veterinarian's confidence and efficiency in undertaking the procedure, but could also indicate that factors other than time, may be important barriers to the routine use of cytology. Previous work also identified challenges to using cytology, and although some did cite consultation duration as a factor, willingness to pay, technical challenges in undertaking the procedure, time delays to results, where not undertaken in the consulting room or where submitted to an external laboratory for analysis and perceived attitudes of clients to the tests were all additionally considered barriers.^27^, ^30^

A novel finding was that veterinarians were often neither familiar with, nor kept in stock, non‐prescription topical chlorhexidine products and lower category oral antimicrobials for cats. These are important therapeutics for veterinary patients presenting with superficial pyoderma and for cats generally. This observation has parallels with a recent Australian study where veterinarians’ prescribing habits were affected by what was available on the consulting room shelf (the ‘dispensary cycle’)^31^ and veterinarians may be unaware of other options if they are not immediately available in their consulting rooms. Furthermore, it was interesting to observe that documentation of the reason for ‘high tier’/HPCIA antimicrobial usage instead of lower tier antimicrobial selection was rarely undertaken. Some veterinarians justified this lack of documentation based on the view they had no suitable non‐tablet alternative therapeutic to prescribe and others noted they did not think it necessary to document the reason for cefovecin use in cats, as they did not consider it ‘off label’. These findings were consistent with previous work, where only 12% of electronic health record entries related to cefovecin usage in cats documented the reason for its use over an alternative antimicrobial and where justification was given it was mostly that there was an inability to orally medicate the cat.^29^ The comment by some veterinarians that documentation of usage of cefovecin was not required as they considered that their usage of cefovecin was not ‘off label’, would appear inconsistent with the RCVS Practice Standard Scheme, the cefovecin datasheet and other antimicrobial guidance.^4^, ^5^, ^32^, ^33^ The core standards of the RCVS Practice Standards Scheme require practices to be able to demonstrate responsible usage of antimicrobials and the basis of their antimicrobial choices, and although not the only way, documentation in the clinical records would provide a transparent way to do this.^32^ Furthermore, although cefovecin usage was not necessarily ‘off label’ given the likely conditions they would be using it for, the cefovecin datasheet warnings and veterinary antimicrobial usage guidance recommend the reservation of cefovecin for bacterial infections that respond poorly to other antimicrobials and based on antimicrobial sensitivity testing.^4^, ^5^, ^33^ These findings could help to explain why previous interventional research on antimicrobial stewardship has shown inconsistent effects on overall and ‘high tier’/HPCIA prescribing rates, since many interventions have not addressed these issues of limited awareness of readily available non‐tablet alternatives, the value of documenting antimicrobial choices in general, or the additional recommendations for ‘higher tier’ antimicrobials. Finally, it was interesting to observe in lower usage practices that they were proud of their antimicrobial performance relative to the group benchmarks. This competitive drive based on a perception of better performance has been reported previously as a potential motivator for improved clinical performance.^34^

While the approach is likely to be widely generalisable, our behaviour‐specific conclusions are less generalisable. One researcher mainly constructed the longlist of behavioural targets, which may have biased the selection process by restricting the list of behaviours that were considered. Furthermore, this single researcher was responsible for visiting practices, collecting data, and coding observations and quotations into theoretical domains and themes. Due to the study time constraints, we did not validate this work either with internal controls or by checking with participants that their meaning and intent had been correctly categorised, so the researcher's biases may have affected the coding. We have provided quotations from a variety of participants to support our findings and interpretations and hope this transparency somewhat mitigates these concerns.

The behavioural targets were complex, the practice environments varied, and we could only attend five practices in the limited time available. As a result, we were unlikely to have reached data saturation. All the practices were in the south of England and there may be regional variations such as socioeconomic pressures, professional networks, and recruitment patterns that mean some relevant observations were missed. This study should therefore not be seen as a comprehensive summary of all possible factors influencing our behavioural targets. The goal was to find common and important factors that are relevant to many practices and can be pragmatically targeted by future interventions.

In summary, this study highlights key behaviours that could be amenable to intervention studies for improved antimicrobial stewardship. It provides a novel approach for identifying behaviours to target for veterinary antimicrobial stewardship intervention studies. Using a structured approach, the study identified common challenges and themes to target for interventions for improved antimicrobial usage and provides a systematic approach for designing behavioural change interventions in a veterinary context.

## AUTHOR CONTRIBUTIONS

Ben Walker performed writing—original draft, conceptualisation, methodology and data curation. Dave Brodbelt and Dan O'Neill performed writing—review and editing, conceptualisation and methodology. Huw Stacey performed writing—review and editing and introduced us to the practices.

## CONFLICT OF INTEREST STATEMENT

The authors declare no conflicts of interest.

## ETHICS STATEMENT

The Royal Veterinary College's Social Sciences Research Ethical Review Board approved the study (URN: SR2021‐0197).

## Supporting information



Supporting Information

Supporting Information

## Data Availability

Due to the sensitive nature of the questions and observations in this study as well as the potential for breach of anonymity from sharing, our raw data must remain confidential and will not be made publicly available.

## References

[1] Dyar OJ , Huttner B , Schouten J , Pulcini C. What is antimicrobial stewardship? Clin Microbiol Infect. 2017;23:793‒798. 28882725 10.1016/j.cmi.2017.08.026

[2] Global and Public Health Group , Emergency Preparedness and Health Protection Policy Directorate. Tackling antimicrobial resistance 2019 to 2024: the UK's 5‐year national action plan. 2019.

[3] Royal College of Veterinary Surgeons . Code of professional conduct for veterinary surgeons and supporting guidance. RCVS; 2022.

[4] British Veterinary Association. Responsible use of antimicrobials. 2019.

[5] Allerton F . Protect me. BSAVA Companion. 2018;2018:8–9.

[6] World Health Organization . Critically important antimicrobials for human medicine, 6th revision. World Health Organization; 2019.

[7] European Medicines Agency . Categorisation of antibiotics in the European Union. Full advice. EMA/CVMP/CHMP/682198/2017. Available from: https://www.ema.europa.eu/en/documents/report/categorisation‐antibiotics‐european‐union‐answer‐request‐european‐commission‐updating‐scientific‐advice‐impact‐public‐health‐and‐animal‐health‐use‐antibiotics‐animals_en.pdf. Accessed 5 June 2024.

[8] World Organisation for Animal Health . WOAH list of antimicrobial agents of veterinary importance. Available from: https://www.woah.org/app/uploads/2021/06/amended‐91gs‐tech‐03‐amr‐working‐group‐report‐en.pdf. Accessed 5 June 2024.

[9] Hubbuch A , Schmitt K , Lehner C , Hartnack S , Schuller S , Schüpbach‐Regula G , et al. Antimicrobial prescriptions in cats in Switzerland before and after the introduction of an online antimicrobial stewardship tool. BMC Vet Res. 2020;16:229. 32620170 10.1186/s12917-020-02447-8 PMC7333330

[10] Walker B , Sánchez‐Vizcaíno F , Barker EN . Effect of an antimicrobial stewardship intervention on the prescribing behaviours of companion animal veterinarians: a pre–post study. Vet Rec. 2022;190:e1485. 35202485 10.1002/vetr.1485

[11] Hopman NEM , Portengen L , Hulscher MEJL , Heederik DJJ , Verheij TJM , Wagenaar JA , et al. Implementation and evaluation of an antimicrobial stewardship programme in companion animal clinics: a stepped‐wedge design intervention study. PLoS One. 2019;14:e0225124. 31738811 10.1371/journal.pone.0225124 PMC6860428

[12] Hardefeldt LY , Hur B , Richards S , Scarborough R , Browning GF , Billman‐Jacobe H , et al. Antimicrobial stewardship in companion animal practice: an implementation trial in 135 general practice veterinary clinics. JAC Antimicrob Resist. 2022;4:dlac015. 35233530 10.1093/jacamr/dlac015 PMC8874133

[13] Singleton DA , Rayner A , Brant B , Smyth S , Noble P‐JM , Radford AD , et al. A randomised controlled trial to reduce highest priority critically important antimicrobial prescription in companion animals. Nat Commun. 2021;12:1593. 33707426 10.1038/s41467-021-21864-3 PMC7952375

[14] Sarrazin S , Vandael F , Van Cleven A , De Graef E, de Rooster H, Dewulf J. The impact of antimicrobial use guidelines on prescription habits in fourteen Flemish small animal practices. Vlaams Diergeneeskd Tijdschr. 2017;86:173‒182.

[15] French SD , Green SE , O'Connor DA , McKenzie JE , Francis JJ , Michie S , et al. Developing theory‐informed behaviour change interventions to implement evidence into practice: a systematic approach using the theoretical domains framework. Implement Sci. 2012;7:38. 22531013 10.1186/1748-5908-7-38 PMC3443064

[16] Tonkin‐Crine S , Walker AS , Butler CC. Contribution of behavioural science to antibiotic stewardship. Br Med J. 2015;350:h3413. 26111947 10.1136/bmj.h3413

[17] Federation of European Companion Animal Veterinary Associations . Advice on responsible use of antimicrobials; 2018.

[18] Weese JS , Giguère S , Guardabassi L , Morley PS , Papich M , Ricciuto DR , et al. ACVIM consensus statement on therapeutic antimicrobial use in animals and antimicrobial resistance. J Vet Intern Med. 2015;29:487–498. 25783842 10.1111/jvim.12562 PMC4895515

[19] Tompson AC , Chandler CIR , Mateus ALP , O'Neill DG , Chang Y‐M , Brodbelt DC. What drives antimicrobial prescribing for companion animals? A mixed‐methods study of UK veterinary clinics. Prevent Vet Med. 2020;183:105117. 10.1016/j.prevetmed.2020.105117 32890918

[20] King C , Smith M , Currie K , Dickson A , Smith F , Davis M , et al. Exploring the behavioural drivers of veterinary surgeon antibiotic prescribing: a qualitative study of companion animal veterinary surgeons in the UK. BMC Vet Res. 2018;14:332. 30404649 10.1186/s12917-018-1646-2 PMC6223057

[21] Presseau J , McCleary N , Lorencatto F , Patey AM , Grimshaw JM , Francis JJ . Action, actor, context, target, time (AACTT): a framework for specifying behaviour. Implement Sci. 2019;14:102. 31806037 10.1186/s13012-019-0951-x PMC6896730

[22] QSR International Pty Ltd . NVivo. 2020. Available from: https://www.qsrinternational.com/nvivo‐qualitative‐data‐analysis‐software/home

[23] Cane J , O'Connor D , Michie S . Validation of the theoretical domains framework for use in behaviour change and implementation research. Implement Sci. 2012;7:37. 22530986 10.1186/1748-5908-7-37 PMC3483008

[24] Viner B. Using audit to improve clinical effectiveness. In Practice. 2009;31:240‒243.

[25] Anthierens S , Tonkin‐Crine S , Douglas E , Fernandez‐Vandellos P , Krawczyk J , Llor C , et al. General practitioners' views on the acceptability and applicability of a web‐based intervention to reduce antibiotic prescribing for acute cough in multiple European countries: a qualitative study prior to a randomised trial. BMC Fam Pract. 2012;13:101. 23110756 10.1186/1471-2296-13-101 PMC3533906

[26] Scarborough RO , Sri AE , Browning GF , Hardefeldt LY , Bailey KE. ‘Brave enough’: a qualitative study of veterinary decisions to withhold or delay antimicrobial treatment in pets. Antibiotics. 2023;12:540. 36978407 10.3390/antibiotics12030540 PMC10044613

[27] Hardefeldt LY , Gilkerson JR , Billman‐Jacobe H , Stevenson MA , Thursky K , Bailey KE , et al. Barriers to and enablers of implementing antimicrobial stewardship programs in veterinary practices. J Vet Intern Med. 2018;32:1092‒1099. 29573053 10.1111/jvim.15083 PMC5980358

[28] Smith M , King C , Davis M , Dickson A , Park J , Smith F , et al. Pet owner and vet interactions: exploring the drivers of AMR. Antimicrob Resist Infect Control. 2018;7:46. 29619213 10.1186/s13756-018-0341-1 PMC5879597

[29] Burke S , Black V , Sánchez‐Vizcaíno F , Radford A , Hibbert A , Tasker S . Use of cefovecin in a UK population of cats attending first‐opinion practices as recorded in electronic health records. J Feline Med Surg. 2017;19(6):687‒692. 27507842 10.1177/1098612X16656706 PMC11128814

[30] Mateus ALP , Brodbelt DC , Barber N , Stärk KDC . Qualitative study of factors associated with antimicrobial usage in seven small animal veterinary practices in the UK. Prev Vet Med. 2014;117(1):68‒78. 25091861 10.1016/j.prevetmed.2014.05.007

[31] Scarborough RO , Bailey KE , Sri AE , Browning GF , Hardefeldt LY . Seeking simplicity, navigating complexity: how veterinarians select an antimicrobial drug, dose, and duration for companion animals. J Vet Intern Med. 2024;38(6):3215‒3234. 39304497 10.1111/jvim.17197 PMC11586579

[32] Royal College of Veterinary Surgeons . Practice standards scheme small animal modules and awards (version 3.3). Core Standard, Section 10.1.28. London: Royal College of Veterinary Surgeons; 2024.

[33] National Office of Animal Health (NOAH) Compendium . Convenia 80 mg/ml powder and solvent for solution for injection for dogs and cats. 2025. Available from: https://www.noahcompendium.co.uk/?id=‐456726. Accessed 22 July 2025.

[34] Gude WT , Brown B , van der Veer SN , Colquhoun HL , Ivers NM , Brehaut JC , et al. Clinical performance comparators in audit and feedback: a review of theory and evidence. Implement Sci. 2019;14:39. 31014352 10.1186/s13012-019-0887-1 PMC6480497

[35] Brissot H , Cervantes S , Guardabassi L , Hibbert A , Lefebvre H , Mateus A , et al. GRAM: guidance for the rational use of antimicrobials. Ceva Santé Animale; 2016.

[36] Weese JS , Blondeau J , Boothe D , Guardabassi LG , Gumley N , Papich M , et al. Vet J. 2019;247:8–25. 30971357 10.1016/j.tvjl.2019.02.008

[37] Hillier A , Lloyd DH , Weese JS , Blondeau JM , Boothe D , Breitschwerdt E , et al. Guidelines for the diagnosis and antimicrobial therapy of canine superficial bacterial folliculitis (Antimicrobial Guidelines Working Group of the International Society for Companion Animal Infectious Diseases). Vet Dermatol. 2014;25:163‐e43. 24720433 10.1111/vde.12118

[38] Servia‐Dopazo M , Taracido‐Trunk M , Figueiras A . Non‐clinical factors determining the prescription of antibiotics by veterinarians: a systematic review. Antibiotics. 2021;10(2):133. 33573109 10.3390/antibiotics10020133 PMC7912449

