# Supplementary material for: A structured, behavioural science approach to the preparation of antimicrobial stewardship interventions for companion animal veterinarians
Source: Vet Rec. 2025 Dec 4;198(12):e521–32. doi: 10.1002/vetr.6016 (PMC13261780; doi:10.1002/vetr.6016)
Supplement: Supplementary file 1 — Supporting Information [file VETR-198--s002.docx]

Supplementary figures


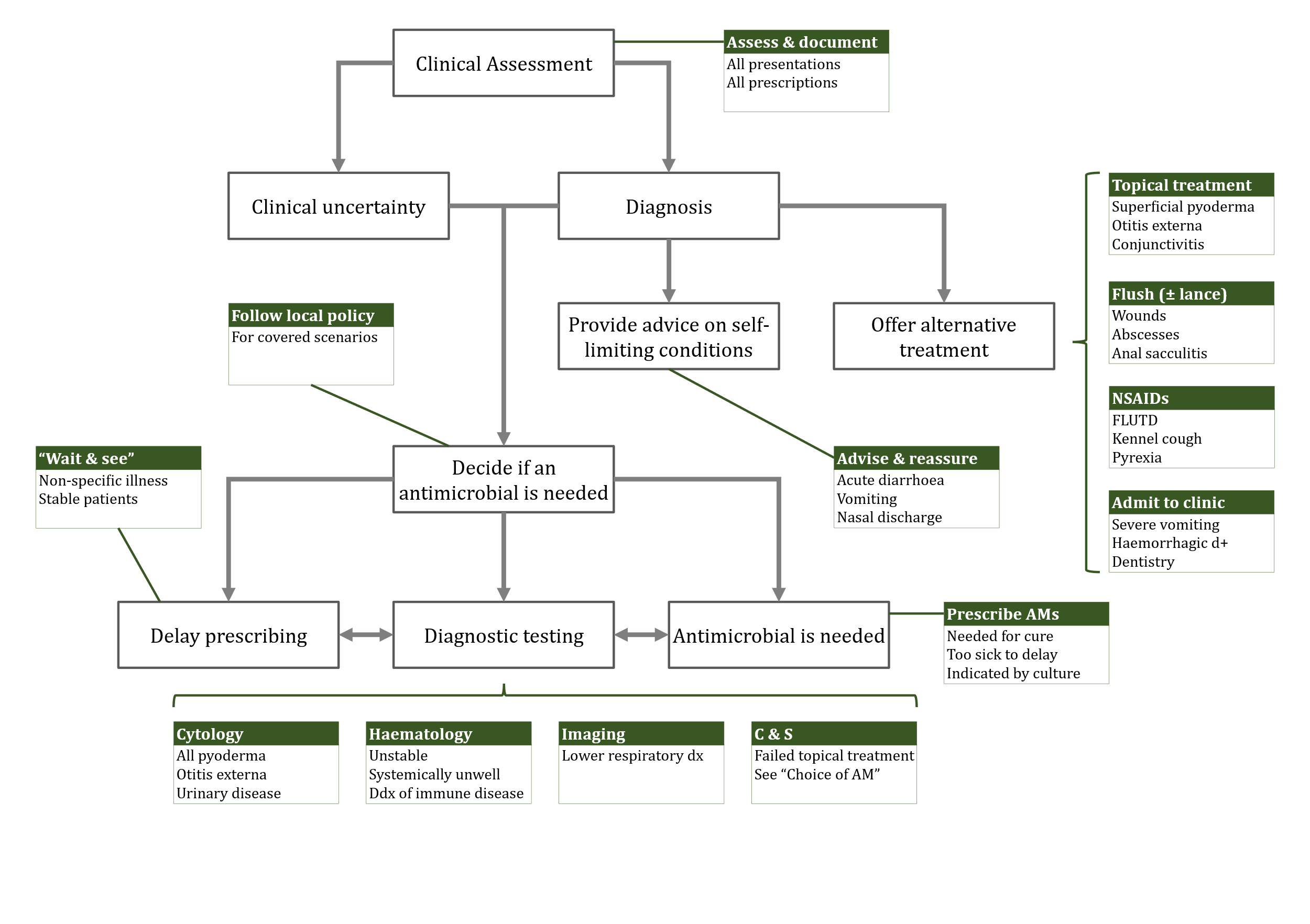


*Supplementary Figure 1: Conceptual map of how antimicrobials are prescribed in veterinary consultations*


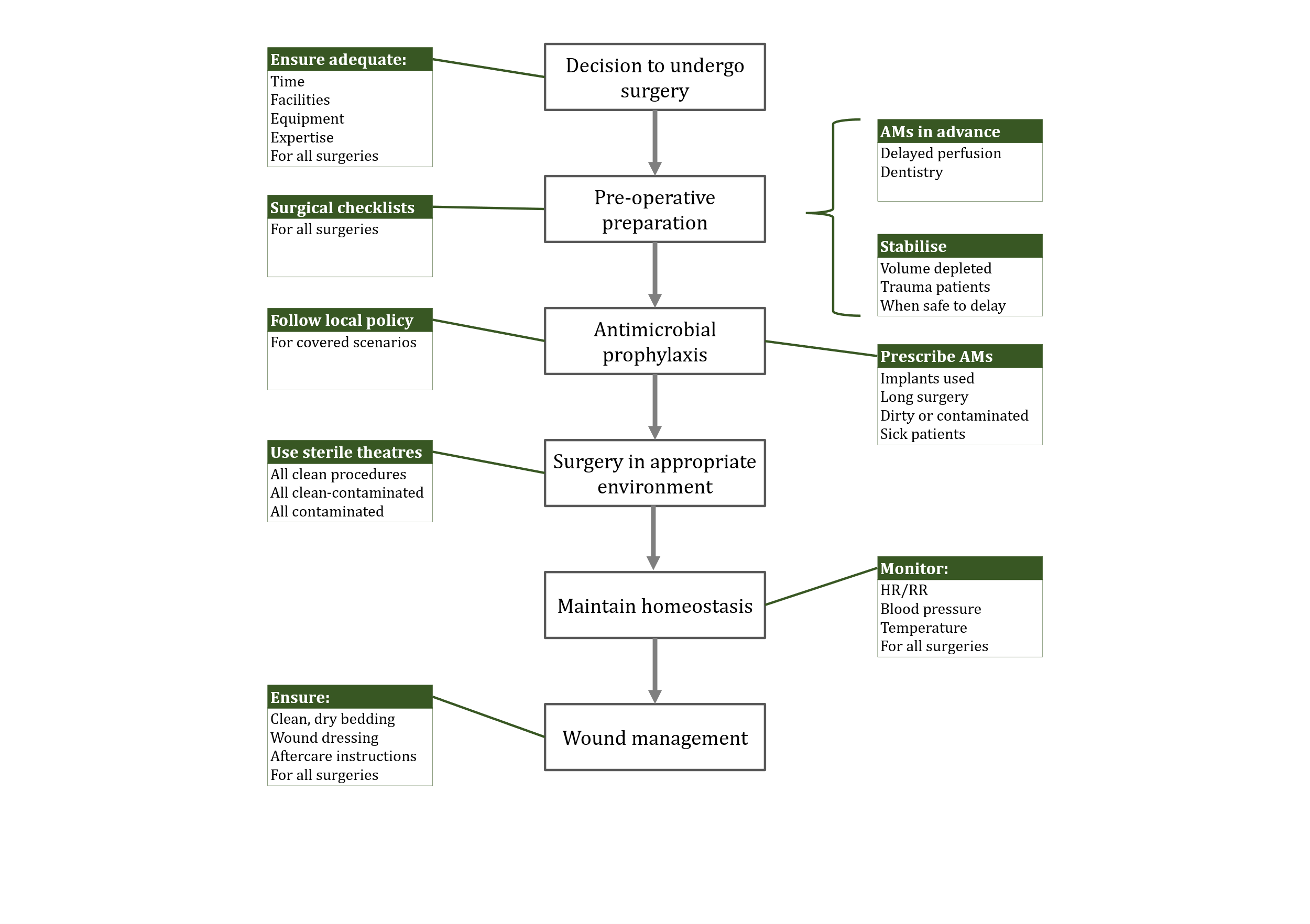


*Supplementary Figure 2: Conceptual map of how antimicrobials are prescribed during veterinary surgery*


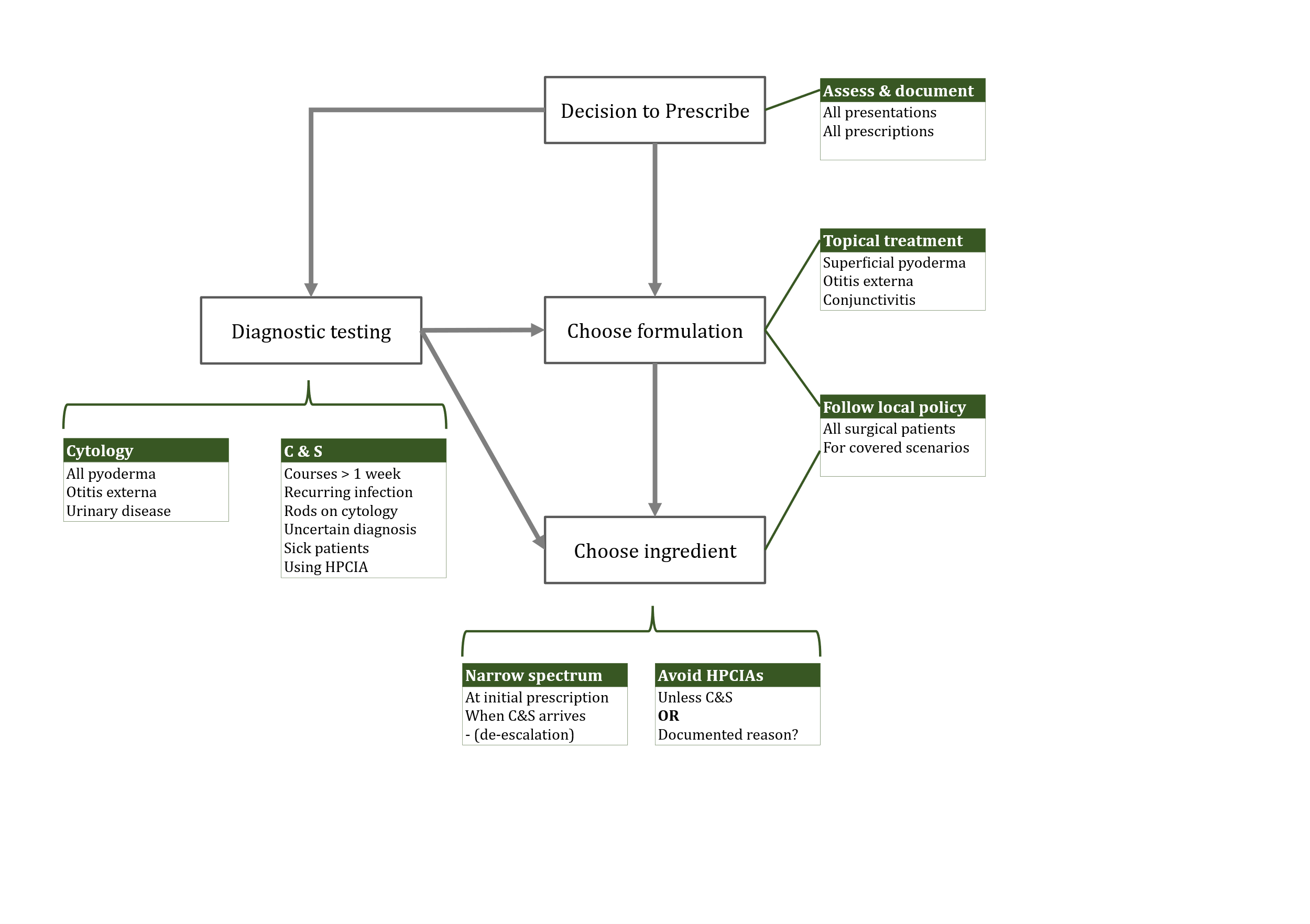


*Supplementary Figure 3: Conceptual map of how antimicrobial products are selected following a decision to prescribe*
