# Supplementary material for: A structured, behavioural science approach to the preparation of antimicrobial stewardship interventions for companion animal veterinarians
Source: Vet Rec. 2025 Dec 4;198(12):e521–32. doi: 10.1002/vetr.6016 (PMC13261780; doi:10.1002/vetr.6016)
Supplement: Supplementary file 2 — Supporting Information [file VETR-198--s001.docx]

# Supplementary Table 1: Antimicrobial Guidelines—Strategic Recommendations

| **Recommendation** | **BVA** | **BSAVA** | **FECAVA** | **ISCAID** | **Ceva** | **Practice Group** |
| --- | --- | --- | --- | --- | --- | --- |
| Reduce overall use | “Minimise use” | “Prescribe only when necessary” | “Avoid unnecessary antimicrobial use” | “Reduced [AM] use in animals might reduce emergence … of AMR” | “Avoid … when they are not necessary” | “Reduce the unnecessary prescribing” |
| Use alternatives | “Use symptomatic relief or topical preparations where appropriate” | “Offer other options” | “Consider use of antiseptics or other agents” | “Not all bacterial infections require … antimicrobials” | • | • |
| Reduce duration | • | “Use the shortest effective course” | • | “Shorter durations of treatment reduce exposure of commensal bacteria [to AMs]” | “Opt for shorter therapeutic course durations whenever possible” | • |
| Reduce use of HPCIAs | “Reserve these [AMs] for clinical conditions that respond poorly  and where [C&S] has been carried out” | “Samples for culture and sensitivity testing should be submitted before starting these” | • | “It is difficult to assign drugs to different tiers with any degree of confidence” | “These … are only recommended if [C&S] demonstrate the need for such a prescription” | “These should only be used when there are no alternative [AMs] authorised  for the target species and indication” |
| *Reserved AMs* | Quinolones  3G cephalosporins  4G cephalosporins  Colistin | Quinolones  3G cephalosporins  4G cephalosporins | • | • | Quinolones  Cefovecin  Chloramphenicol  Aminoglycosides  Macrolides  Metronidazole | Quinolones  3G cephalosporins  4G cephalosporins  Colistin |

| *Restricted AMs* | • | Glycopeptides  Carbapenems  Oxazolidines  “[AMs] with restricted use in human medicine” | • | • | Glycopeptides  Carbapenems  Monobactams  Oxazolidines  Lipopeptides  Riminofenazines | • |
| --- | --- | --- | --- | --- | --- | --- |
| Use narrow spectrum | “Use narrow spectrum  antimicrobials where possible” | “Employ narrow spectrum” | “If indicated, change treatment according to laboratory results and if possible to an antimicrobial with the narrowest spectrum” | “There are no clear and logical definitions for these terms” | “The choice should fall on the drug that has the least possible impact on selection of multidrug-resistant bacteria” | • |
| Use C&S | “Bacterial culture and sensitivity must be determined whenever possible” | “Culture appropriately” | “Take samples for culture and susceptibility testing” | “The Committee strongly supports the use of bacterial culture” | “Cytological and microbiological  diagnosis are requirements for appropriate antimicrobial therapy” | “The cost and time involved in [C&S] testing can present a challenge in general practice. Here are some top tips” |
| Adequate dosing | “Avoid underdosing” | “Avoid underdosing” | • | “Label dose [and] dosing interval … are not always consistent with current principles of … use” | “Underdosing and irregular administration intervals should be avoided” | • |

# Supplementary Table 2: Antimicrobial Guidelines—System Recommendations

| **Recommendation** | **BVA** | **BSAVA** | **FECAVA** | **ISCAID** | **Ceva** | **Practice Group** |
| --- | --- | --- | --- | --- | --- | --- |
| Local prescribing policy | “Create practice-based protocols for common infections” | “Tailor your practice policy” | “Local resistance patterns have to be taken into consideration” | • | “Adopt written infection control protocols” | “Partners [can] record their own local treatment protocols” |
| Auditing | “Regularly assess antimicrobial use” | “Audit your own antibacterial use” | • | • | “Good record keeping and effective use of clinical audit are a professional responsibility” | “Make sure that you review and discuss the reports” |
| Client handouts | • | “Use non-prescription forms” | “Use printed documentation (leaflets, posters) & face-to-face communication” | • | “[Provide] detailed written instructions to pet owners” | “The client leaflets and poster … will help to support and justify your clinical decision making” |
| Stewardship champions | • | “The antibiotic guardian(s) of this practice is/are” | • | • | “Appoint an infection control champion” | “The JVP or a … colleague needs to act as the Antibiotics STAR” |
| Training | “Know how antimicrobials work and their pharmacodynamic properties” | • | • | • | “Train and encourage all staff to follow infection control guidelines” | • |
| Dispensary | • | • | • | • | • | “Move all of your antibiotics in to a single cupboard” |

# Supplementary Table 3: Antimicrobial Guidelines—Scenario Recommendations

| **Scenario** | **BVA** | **BSAVA** | **FECAVA** | **ISCAID** | **Ceva** | **Practice Group** |
| --- | --- | --- | --- | --- | --- | --- |
| Canine superficial pyoderma |  |  |  |  |  |  |
| *Alternative to AM* | • | Topical treatment | Topical treatment | Topical treatment | Topical treatment | Topical treatment |
| *Empiric treatment* | • | Clindamycin  Cefalexin  Amoxiclav  TMPS  3+ weeks at high dose | Clindamycin  1G cephalosporins  Amoxiclav  TMPS  Until resolved | Clindamycin  Lincamycin  1G cephalosporins  Amoxiclav  TMPS  ± cefovecin  3+ weeks | Amoxiclav  Cefalexin  Cefadroxil  Clindamycin  3+ weeks | Cephalosporin |
| Canine acute diarrhoea |  |  |  |  |  |  |
| *Alternative to AM* | • | No treatment | No treatment | • | No treatment | • |
| *Systemic signs* | • | Amoxiclav  Cefalexin | • | • | As per sepsis | • |
| Canine uncomplicated UTI |  |  |  |  |  |  |
| *Empiric treatment* | • | Amoxicillin  Amoxiclav  TMPS  7–10 days | Amoxicillin  TMPS  Amoxicillin q8h | Amoxicillin  Amoxiclav  TMPS  3–5 days | TMPS  Amoxicillin (cocci)  Amoxiclav (bacilli)  7 days | Amoxiclav  TMPS  Cephalosporin |
| Canine acute prostatitis |  |  |  |  |  |  |
| *Empiric treatment* | • | Quinolones  TMPS  4–6 weeks | • | TMPS  Quinolones  4 weeks | TMPS  3–4 weeks | TMPS  Quinolones |

| **Scenario** | **BVA** | **BSAVA** | **FECAVA** | **ISCAID** | **Ceva** | **Practice Group** |
| --- | --- | --- | --- | --- | --- | --- |
| Feline cat bite abscesses |  |  |  |  |  |  |
| *Systemically well* | • | Topical treatment  ± amoxiclav (7 days) | Topical treatment | • | Topical treatment | Clindamycin  Cephalosporin |
| *Systemic signs* | • | Clindamycin (cocci)  Cefalexin (cocci)  Amoxiclav (cocci)  TMPS (cocci)  Quinolones (rods) | • | • | Amoxicillin  Amoxiclav  Cefalexin  Cefadroxil  4–5 days | • |
| Anal sac disorder |  |  |  |  |  |  |
| *When to give AM* | • | Abscessation + cellulitis | Abscessation + severe damage  ± fever | • | Abscessation + systemically unwell | Abscessation |
| *Empiric treatment* | • | TMPS  Amoxiclav | TMPS  Amoxiclav (cats) | • | Amoxiclav  Cefalexin | TMPS  Cephalosporin |
| Otitis externa  (No ruptured TM) |  | Topical treatment | Antiseptics  Topical treatment |  | Topical treatment |  |
| *Rods* | • | Framycetin  Gentamicin  Polymyxin B | Follow cascade | • | Polymyxin B  Silver sulfadiazine | • |
| *Cocci* | • | Florfenicol  Fusidic acid/framycetin  Polymyxin B/miconazole | Follow cascade | • | Neomycin  Fusidic acid  Framycetin  Florfenicol | • |

| Sepsis | • | Quinolone AND (clindamycin OR amoxiclav OR  ampicillin with metronidazole/amikacin) | Quinolone AND (clindamycin OR penicillin G OR ampicillin OR amoxicillin) | • | Quinolone AND (amoxicillin OR amoxiclav OR clindamycin)  4–6 weeks | Amoxiclav OR  Quinolone AND (clindamycin OR metronidazole) |
| --- | --- | --- | --- | --- | --- | --- |
| Surgical prophylaxis |  |  |  |  |  |  |
| *When indicated* | • | >90 minutes  Implants  Intestinal or urinary tract  CNS  Contamination  Debilitated patients | • | • | >90 minutes  Implants  Contamination | >90 minutes  Implants  Intestinal tract  Dentistry  CNS  Contamination  Debilitated patients |
| *AM choice* | • | Amoxiclav  Cefuroxime  ± metronidazole | • | • | Amoxiclav  ± metronidazole | Amoxiclav  1G cephalosporins  ± metronidazole |
